# Impact of sand media continuous drying and rewetting cyclic on nutrients transformation performance from reclaimed wastewater effluent at soil aquifer treatment

**DOI:** 10.1038/s41598-024-58787-0

**Published:** 2024-04-05

**Authors:** Abdalkarim S. Gharbia, Balázs Zákányi, Márton Tóth

**Affiliations:** Faculty of Earth Science and Engineering, Institute of Environmental Management, University of Miskolc, Miskolc, Hungary

**Keywords:** Soil aquifer treatment, Reclaimed wastewater, Wet and dry cycles, Geochemistry, Pollution remediation

## Abstract

Reusing reclaimed wastewater became a practical resource for water utilization in groundwater recharge and irrigation activities. However, the quality of reclaimed wastewater needs improvement to meet the environmental regulations and reduce contamination risks. A laboratory-scale study simulated a soil aquifer treatment (SAT) system, exploring the synergistic effects of wet and dry cycles alongside key physicochemical parameters on pollutant removal efficiency using a glass column filled with quartz sand as the filtration medium. The investigation focused on the cyclic wetting and drying phases to unravel their impact on removing NH_4_^+^, NO_3_^−^, and PO_4_^3−^. The synthetic wastewater introduced into the system exhibited varying pollutant concentrations during wet and dry periods, influenced by dynamic soil water content (WC%), pH, dissolved oxygen (DO), and oxidation–reduction potential (ORP). The high removal rates of 93% for PO_4_^3−^ and 43% for Total N_2_ demonstrate the system’s capability to reduce concentrations significantly under dynamic alternating between wet and dry conditions. Results unveiled that the wet period consistently yielded higher removal rates for N_2_ species. Interestingly, for PO_4_^3−^, the dry periods demonstrated a higher removal efficiency. Moreover, the study identified an average NO_3_^−^ production during the experimental phases as a byproduct of nitrification. The average NO_3_^−^ production in wet periods was 2.5 mg/L, whereas it slightly decreased to 2.2 mg/L in dry periods. These findings underscore the nuanced influence of wet and dry conditions on specific pollutants within SAT systems. Applying the logistic regression model and principal component analysis demonstrated the statistical significance of WC, pH, DO, and ORP in predicting wet/dry conditions, providing quantitative insights into their influential roles on the nutrient dynamic concentrations. This study contributes valuable data to our understanding of SAT systems, offering practical implications for designing and implementing sustainable wastewater treatment practices and pollution management across diverse environmental contexts.

## Introduction

Insufficient water resources in arid and semi-arid communities present a significant challenge for sustaining water demand across various sectors^1^. To address this challenge, wastewater reuse in agriculture and groundwater recharge, after partial treatment, has emerged as an alternative water source^2^. However, for treated wastewater effluents to become beneficial resources for groundwater recharge and agricultural activities, they must adhere to environmental standards and regulations^3^. The need for compliance underscores the necessity to enhance reclaimed wastewater quality. Natural treatment technologies, such as soil aquifer treatment (SAT), integrate soil with aquifers to improve the quality of infiltrated wastewater, acting as a supplementary treatment application^4^. In SAT systems, complex physical, chemical, and biological interactions occur, including biodegradation, ion exchange, adsorption, precipitation, and redox reactions^5^. The efficiency of the treatment system is influenced by various factors, such as soil structure, infiltrated water quality, hydraulic conditions, microbial community, water retention time, and drying and wetting cycles^6^. The vadose layer, extending from the ground surface to the groundwater, is crucial in protecting groundwater from surface pollutants leaching^7^. Soil, the primary medium for pollutant filtration, defines pollutant destinies, transformations, and reactions, with the perfect media exhibiting rapid infiltration and complete decontamination^8^.

The continuous variability of saturation degree in the vadose zone influences contaminant mobility and fate behavior under biogeochemical processes, affecting parameters such as microbial cultures, the water content in soil pore spaces, and the availability of dissolved oxygen^9^. Hydraulic conditions, especially continuous drying and rewetting, are critical factors affecting SAT systems' performance and removal capacity^10^. Interactions between drying and rewetting hydraulic conditions and decontamination procedures contribute to clogging points, impacting infiltration rates^11^. The drying and rewetting cycle, fundamental to SAT, allows for rebuilding infiltration capacity and diffusing oxygen into clogged soil layers, relying on complex biogeochemical operations during wastewater infiltration in the vadose zone^12^. Transitioning to the broader context of soil aquifer treatment (SAT) systems, these systems are widely employed for wastewater treatment, offering a natural and sustainable approach to pollutant removal before water is recharged into aquifers or reused for various purposes^13^. In SAT systems, the physical, chemical, and biological processes within the soil matrix and aquifer are crucial in transforming and removing pollutants^4^. Among the various factors influencing pollutant behavior in SAT systems, the dynamics of drying and wetting cycles have been recognized as essential drivers of pollutant fate and transport^14^.

In SAT systems, the fluctuating wet and dry cycles profoundly affect pollutant behavior and removal efficiency. These observations are governed by fundamental hydrogeology and environmental management theories, demonstrating the intricate interplay between hydraulic conductivity, biogeochemical processes, and pollutant dynamics within porous media. The wetting and drying cycles in SAT systems are supported by variations in hydraulic conductivity, influenced by soil moisture content^15^. During wet periods, increased soil moisture enhances hydraulic conductivity, facilitating the movement of water and dissolved contaminants through the porous media^8^. This promotes the release of certain pollutants into the surrounding environment. Conversely, during dry periods, reduced soil moisture decreases hydraulic conductivity, leading to enhanced retention and immobilization of contaminants within the porous media through processes such as adsorption onto soil particles and microbial degradation^16^. While our experimental observations provide valuable insights, a deeper understanding can be gained by relating our findings to fundamental theories governing hydraulic and biological processes within such systems. The BIO_PORE model, as Samsó and Garcia^17^ outlined, offers a mathematical framework for simulating biofilm growth and water quality improvement in porous media, such as constructed wetlands. Similarly, the Cartridge Theory, as discussed in Samsó and García^18^, describes the functioning of horizontal subsurface flow constructed wetlands, providing insights into pollutant removal mechanisms.

Understanding the effect of drying and wetting cycles on synthetic wastewater pollutants in SAT systems is crucial for optimizing system design, operation, and pollutant removal efficiency. These cycles induce movement and redistribution of contaminants within the soil aquifer system, influencing their long-term fate and persistence^19^. Wetting events during these cycles carry pollutants deeper into the subsurface, influenced by solubility, sorption affinity, and soil hydraulic properties^20^. Conversely, drying periods may lead to pollutant accumulation near the surface or specific soil horizons, impacting pollutant transport dynamics^21^.

Moreover, drying and wetting cycles create dynamic conditions influencing microbial activity and biodegradation of pollutants^22^. Wetting events provide favorable conditions for microbial growth, promoting activity in pollutant degradation and transformation processes^23^. Adequate moisture during wetting enhances dissolved oxygen availability, facilitating aerobic degradation pathways. Conversely, drying periods may limit microbial activity due to reduced moisture and oxygen, potentially decreasing pollutant degradation rates^24^. The transition between aerobic and anaerobic conditions during drying may promote different pollutant transformations, affecting overall removal efficiency^25^.

Drying and wetting cycles significantly influence the physical properties of the soil, including its structure, porosity, and hydraulic conductivity^26^. Extended drying periods can lead to soil compaction, shrinkage, and the development of cracks, influencing water movement and pollutant transport. Conversely, wetting events can help restore soil structure and porosity, enhancing treatment efficiency^27^. These cycles also affect the adsorption and desorption of pollutants onto soil particles^28^. During wetting, pollutants may be adsorbed onto soil surfaces, reducing their mobility, while drying periods can promote desorption, potentially increasing pollutant mobility^29^. The cycling of wetting and drying further influences the leaching and transport of pollutants within the soil aquifer system^30^. Wetting events can mobilize pollutants, increasing the potential for downward transport and leaching into the groundwater. Conversely, drying periods may reduce leaching by promoting pollutant retention within the soil matrix, although cracks or macropores formed during drying events can create preferential flow paths, impacting pollutant transport and system efficiency^31^.

Several studies extensively explore the efficacy of SAT systems in mitigating various pollutants from partially treated wastewater, covering a broad types and origins of contaminants. However, a notable gap holds in our understanding of the nuanced dynamics of hydraulic and operational conditions governing the behavior of specific nutrients, particularly Ammonium (NH_4_^+^), Nitrate (NO_3_^−^), and Phosphate (PO_4_^3−^), within SAT systems amidst alternating dry and wetting cycles. This gap is essential due to its direct entanglements for sustainable water management practices. It provides actionable insights for SAT systems optimization, design, and operation by focusing sharply on the influence of dynamic hydraulic and operational conditions, such as drying and wetting cycles, on the nutrient mechanisms governing fate and transport dynamics relating to the transformation and removal efficiency of NH_4_^+^, NO_3_^−^, and PO_4_^3−^ pollutants in SAT systems.

Therefore, this investigation study aims to explore the influence of dynamics hydraulic operation conditions, specifically the alternating wetting and drying cycles, on the transformation and removal efficiency of NH_4_^+^, NO_3_^−^ PO_4_^3−^ as nutrients wastewater pollutants in a simulated soil aquifer treatment (SAT) system. To address this primary objective, the study questioned: How do continuous short-term durations of wetting and drying cycles, representative of dynamic hydraulic conditions in SAT systems, influence the transformation and removal capacity of NH_4_^+^, NO_3_^−^ and PO_4_^3−^ pollutants in reclaimed synthetic wastewater? Also, it hypothesized that alternating wetting and drying cycles would significantly impact the fate and behavior of NH_4_^+^, NO_3_^−^ and PO_4_^3−^ pollutants within infiltrated in the SAT system. Specifically, wetting events are anticipated to motivate pollutant mobilization and microbial activity, enhancing pollutant degradation and transformation processes. Conversely, drying periods are expected to result in the accumulation of pollutants near the surface or in soil horizons, consequently influencing pollutant transport dynamics and overall removal efficiency.

## Materials and methods

A lab-scale glass column with a 3 cm inner diameter and 40 cm depth was utilized to simulate a soil aquifer treatment (SAT) system by incorporating quartz sand soil as the filtration media up to 30 cm of depth in a controlled laboratory setting with soil physical characteristics, as presented in Table 1.

**Table 1:** Physical characteristics of packing quartz sand.

Property		Quartz sand
Particle size (mm)		0.50–0.125
Porosity		0.43
Bulk density (g/cm^3^)	Dry	1.48
	Wet	1.81

A synthetic wastewater mixture was designed by mixing 176.5 mg of KH_2_PO_4_, 104 mg NH_4_CL, 200 mg yeast extract, and 200 mg peptone into 1 L measuring flask with refilling and combining it with distilled water to have 35 mg/L of NH_4_^+^, and 20 mg/L of PO_4_^3−^ with saving a high efficacy rate for nitrification by using peptone and yeast extract as source of nutrients and coenzymes factors for nitrifying bacteria. Synthetic wastewater solution was introduced into the column through a peristaltic pump, following a controlled infiltration rate of 0.5 mL/min. The total daily amount of infiltrated synthetic wastewater was 200 mL/day, into a wetting period of 8 h and a drying period of 16 h. This wetting and drying cycle was repeated continuously for 4 days. Using the direct reading spectrophotometer device, the experiment collected samples at regular intervals to measure the concentration of pollutants, including NH_4_^+^, NO_3_^−^, and PO_4_^3−^. Additionally, physiochemical parameters such as soil water content (WC%), pH, dissolved oxygen (DO), and oxidation–reduction potential (ORP) were measured using HACH meters for each collected sample. In conjunction with these measurements, a comprehensive series of statistical analyses were conducted to explore the relationships and interactions between pollutant concentrations and the varying wet and dry conditions. All plots, statistical analyses, and visualizations were executed using the R programming language.

This study implemented rigorous standard operating procedures as control conditions to ensure consistency and reliability throughout the experimental, measurements, and analysis processes. These procedures were developed and followed meticulously to maintain uniformity in experimental conditions, data collection and analysis procedures. Sample collection and handling: Standardized protocols were established for collecting soil samples from the experimental columns at designated intervals. Samples were handled with care and pretreated to prevent contamination and ensure the integrity of the collected data. Instruments calibration: Before each measurement session, all instruments used for physicochemical parameters and concentration measurements (pH, DO, ORP meters, and spectrophotometer) were calibrated according to manufacturer specifications. Calibration records were maintained to track instrument performance and ensure accuracy in measurements. Experimental setup and operation: Detailed instructions were followed for setting up the experimental columns, including the precise measurement of uniform packing of quartz sand soil and the controlled introduction of synthetic wastewater. Operating parameters such as flow rate, wetting and drying cycles, and sampling intervals were strictly adhered to throughout the experiment.

Replication for accuracy: The experimental procedures were iteratively conducted three times to enhance reliability and confidence in the obtained results. This replication process involved repeating the entire experiment under identical conditions simultaneously. By experimenting multiple times, we aimed to minimize the influence of random variability and increase the robustness of our findings, illustrating the 95% confidence intervals. Adhering to these standardized protocols allowed us to confidently interpret the results of our study and draw meaningful conclusions about the impact of synthetic wastewater on pollutant concentrations in the soil column.

The physiochemical parameters (pH, DO, and ORP) are used as wet/dry conditions factors, considered the main changeable parameters for the whole system under continuous alternating of dry and wet periods. A logistic regression model explored these parameters’ significant relation with wet/dry time conditions. The rationale for choosing logistic regression as the primary analytical approach is elaborated upon to investigate the significance of physicochemical parameters (pH, DO, and ORP) concerning logistic regression is well-suited for analyzing categorical dependent variables, such as wet and dry conditions, and assessing the likelihood of specific outcomes based on predictor variables. In this study, wet and dry conditions are binary categories, making logistic regression an appropriate method to evaluate the influence of physicochemical parameters on the probability of each condition occurring.

In addition, the principal component analysis (PCA) was employed to investigate the effect of continuous dynamic alternating of wet and dry conditions on NH_4_^+^, NO_3_^−^, and PO_4_^3−^ concentrations in soil aquifer treatment. Principal Component Analysis (PCA) is a statistical technique used to analyze multivariate data and identify patterns by reducing its dimensionality while preserving most of the variability in the original dataset^32^. PCA transforms the original variables into a new set of orthogonal variables called principal components. These principal components are linear combinations of the original variables^33^. PCA is widely used in several fields, including water quality, wastewater treatment, and environmental applications, to investigate relationships among variables, detect patterns, and identify essential parameters driving variability in complex datasets^34^. By summarizing information across multiple correlated variables, PCA facilitates data interpretation and visualization, aiding in identifying key factors influencing the system under study.

PCA use in the analysis is related to the dataset likely containing multiple correlated variables (e.g., WC, pH, DO, ORP, NH_4_^+^, NO_3_^−^, PO_4_^3−^), challenging defining and underlying patterns and relationships. PCA reduces dimensionality by condensing data from these variables into fewer uncorrelated principal components. Also, PCA enables the identification of controlled factors driving variability in the dataset by transforming the original variables into principal components. This is particularly relevant in understanding the impact of dry and wet conditions on the concentrations of NH_4_^+^, NO_3_^−^, and PO_4_^3−^. PCA also provides statistical summaries, such as eigenvalues and contributions of variables to principal components, which offer insights into the relative importance of variables and their contributions to variability in the dataset.

Breakthrough curves provide insights into the total mass removed by the infiltrate media, denoted as q_total_, under specific conditions over time (t)^35^. This is calculated using Eq. (1):

1 $${q}_{total}= \frac{QA}{1000}= \frac{Q}{1000} {\int }_{0}^{{t}_{total}}{C}_{ad} dt$$

Where: Q represents the infiltration rate (mL/min), A is the area under the breakthrough curve, t is the total duration time of infiltration (min), and C_ad_ is the concentration (mg/L).

The total pollutants removal capacity rate (R%) can be calculated from the ratio of uptake pollutant mass (q_total_) to the total amount of pollutants infiltrated into the sand column as^36^:

2 $$R\mathrm{\%}= \frac{1000* {q}_{total}}{{C}_{o}*Q*{t}_{total}}*100\mathrm{\%}$$

## Results and discussions

**Figure 1:**
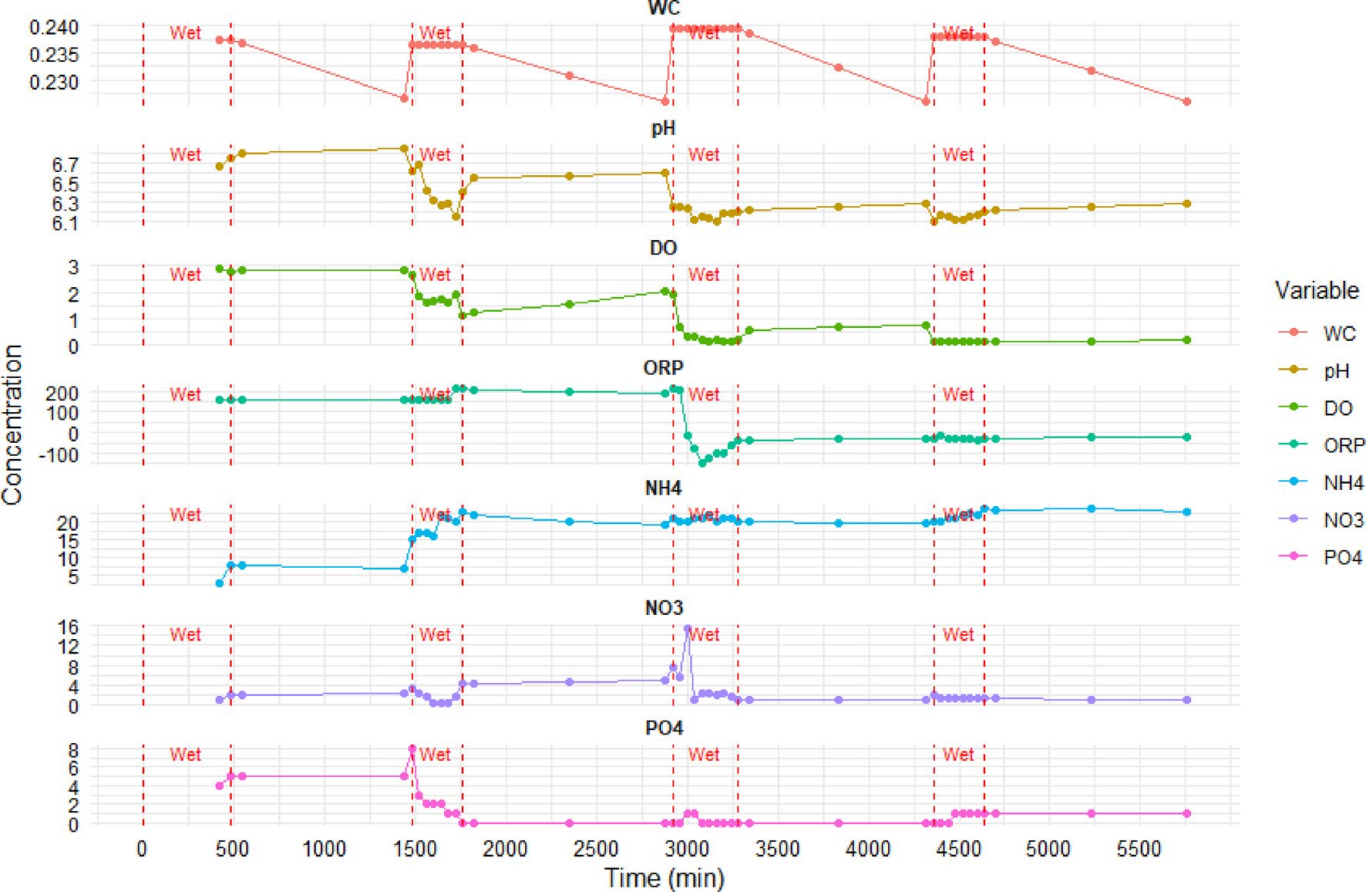
Physiochemical parameters and nutrients (NH_4_^+^, NO_3_^−^, PO_4_^3−^) concentration under wet and dry times.

Several physicochemical parameters, such as soil water content (WC%), pH, dissolved oxygen (DO), and oxidation–reduction potential (ORP), as well as the NH_4_^+^, NO_3_^−^, and PO_4_^3−^ concentrations, were determined for each sample collected under the wet and dry times, as shown in Fig. 1. Furthermore, a descriptive analysis performed to comprehensively describe the experimental results highlights varying degrees of variability across the parameters, as shown in Table 2.

**Table 2:** Parameters descriptive statistical summary.

Variable	Min	Mean	Max	StdDev
WC	0.2258	0.2362	0.2396	0.00405
pH	6.1	6.32	6.85	0.21484
DO	0.1	0.973	2.91	0.96905
ORP	− 142.7	51.649	213.8	117.865
NH_4_^+^	3	19.126	23.7	4.76471
NO_3_^−^	0.583	2.5168	15.4	2.62043
PO_4_^3−^	0	1.2308	8	1.84193

WC values are consistently close to the mean with minimal variability. pH levels vary moderately, ranging from slightly acidic to slightly alkaline. DO levels have significant variability, ranging from very low to relatively high. ORP values show substantial variability, suggesting diverse oxidative states in the water. NH_4_^+^ concentrations display moderate variability, while NO_3_^−^ levels exhibit considerable fluctuations. PO_4_^3−^ concentrations show moderate variability.

These physiochemical parameters were selected to investigate how drying and wetting conditions influenced pollutant behavior and removal efficiency. The water content (WC%), representing the variation of moisture content in the soil filtration media, plays a pivotal role in the behavior of synthetic wastewater pollutants within the soil aquifer treatment system as synthetic wastewater infiltrates the system during wetting, WC% increases, enhancing the transport and interaction between contaminants and the soil matrix. Conversely, in the drying phase, WC% decreases, restricting pollutant mobility. Figure 1—WC illustrates the dynamic shifts in WC% during wet and dry periods. The wetting phase elevated WC% to 23.75%, then declined to 22.66% during the subsequent drying phase. This pattern continued similarly during the experimental days, reflecting the natural dynamics of wetting and drying. These fluctuations have significant implications for pollutant behavior, mobilization affecting diffusion, adsorption, and microbial activity within the soil aquifer treatment system, thereby influencing pollutant transformation and removal.

Figure 1—pH illustrates the pH trendy variations in the sand column of the simulated SAT system soil aquifer treatment system during wet and dry periods. Initially, the pH of the sand infiltrate media was 6.85, while the synthetic wastewater had a pH of 6.5. On the first day, the pH dropped slightly to 6.75 during the wetting phase, potentially due to acidic components in the synthetic wastewater, equilibrium balance and microbial activity. As the system transitioned to the drying period, the pH increased to 6.85, possibly resulting from the evaporation of water, concentrating alkaline compounds. A similar pattern of pH changes continued on the second, third, and fourth days, reflecting the dynamic nature of the system. Factors like acidic components in the wastewater and microbial activity influence these fluctuations. Generally, the decreasing pH level is evidence of an active nitrification process, which produces H^+^ protons as a byproduct from NH_4_^+^ oxidation that increases the system’s acidity.

Dissolved oxygen (DO) represents the amount of oxygen in the wastewater. It is an essential factor for the activity of aerobic microorganisms responsible for biodegradation and removal of organic pollutants. The changes observed in the dissolved oxygen (DO) concentrations during the wet and dry periods in Fig. 1—DO can be attributed to several factors related to microbial activity and the physicochemical processes occurring in the sand column.

The dissolved oxygen (DO) concentration dynamics in the synthetic wastewater infiltrated sand column during wet and dry periods reveal a complex interplay of factors. Initially, the DO concentration was 2.91 mg/L on the first day during the wetting period, gradually declining to 2.78 mg/L by the end of the wet phase. This decrease can be attributed to microbial respiration and the oxygen consumption by microorganisms within the sand column. These microorganisms metabolize organic matter, leading to a reduction in DO levels. In contrast, during the subsequent dry period, the DO concentration increased to 2.81 mg/L at the beginning of the drying phase, gradually rising to 2.86 mg/L by the end. This increase in DO during drying is due to limited water availability, allowing for enhanced oxygen transfer and replenishment. As water content decreases, the improved surface area for gas exchange promotes oxygen diffusion into the system. A similar pattern was observed on the second, third, and fourth days: DO concentration dropped during wetting time, reaching lows attributed to increased microbial activity and organic matter degradation with oxygen unavailability. During the subsequent dry period, DO concentration gradually improved due to enhanced oxygen transfer as water content decreased and the system became less saturated. The fluctuations in DO levels result from factors such as oxygen consumption during wetting, limited oxygen transfer during wetting, oxygen replenishment during drying, and microbial activity.

Oxidation–reduction potential (ORP) measures a solution's electron transfer potential and indicates the redox conditions. ORP influences the oxidation and reduction reactions that occur during the degradation and transformation of pollutants. During wetting, the ORP may change due to different oxidizing or reducing agents in synthetic wastewater. These variations in ORP can impact the rates of pollutant degradation and the controlled conditions for transformation. The changes observed in the oxidation–reduction potential (ORP) values during the wet and dry periods, as shown in Fig. 1—ORP, reflect the redox reactions and the shifting balance between oxidizing and reducing conditions in the sand column.

The oxidation–reduction potential (ORP) within the sand column exhibited dynamic changes during wet and dry periods, reflecting the interplay of multiple factors. During the wetting phase, the first and second days indicate more oxidizing conditions due to enhanced oxygen availability and oxidation reactions. In the dry period, ORP decreased. On the third day, they presented complex ORP changes. During wetting, ORP initially increased, then suddenly dropped to − 13.6 mV, signifying a significant shift towards reducing conditions. This sharp decrease resulted from oxygen depletion, likely being unavailable in the system, and the presence of reduced species, likely stemming from microbial activity. Oxygen consumption by microorganisms can exceed supply, prompting anaerobic respiration and the accumulation of reduced compounds, leading to a highly reducing environment. During drying, ORP gradually increased, suggesting a transition towards less-reducing conditions. On the fourth day, ORP remained stable during wetting, indicating persistent reducing conditions. In the subsequent drying period, ORP gradually increased, shifting towards less-reducing conditions. These ORP changes are influenced by factors such as oxygen availability, microbial activity, and the presence of organic matter. Wetting brings in dissolved oxygen from the infiltrated wastewater, increasing oxygen availability and leading to more oxidizing conditions. Microbial activity and organic matter breakdown then contribute to decreasing ORP during wetting. In the drying phase, limited oxygen leads to more reducing conditions as aerobic microbial activity wanes. Microbial activity and the interplay between oxidation and reduction reactions are critical determinants of ORP dynamics during wet and dry periods.

Furthermore, the physiochemical parameters in Fig. 1 show the breakthrough curves for NH_4_^+^, NO_3_^−^, and PO_4_^3−^. The breakthrough curve for NH_4_^+^ through the sand column during the wet and dry periods over a 4 day column experiment with an initial concentration of 35 mg/L is shown in Fig. 1—NH_4_. NH_4_^+^ concentration changes in a sand column wastewater treatment system over 4 days, involving alternating wet and dry cycles. Notable patterns include NH_4_^+^ concentration increases during wetting periods, attributed to NH_4_^+^ release from synthetic wastewater, followed by slight decreases during drying periods, likely due to increased adsorption onto sand particles and potential microbial processes. The findings indicate the effective removal of NH_4_^+^ from the wastewater, with the sand column successfully adsorbing and degrading a substantial portion of the initial NH_4_^+^ concentration, which commenced at 35 mg/L. The removal mechanisms involved physical adsorption onto sand particles and potential microbial processes leading to NH_4_^+^ degradation or transformation.

Several studies have explored NH_4_^+^ dynamics in SAT systems under static conditions, elucidating mechanisms such as adsorption onto sand particles, microbial degradation, and nitrification processes. For instance, Subari, Abdullah^37^ and Bori Akadar, Bourioug^38^ demonstrated the effectiveness of sand in removing NH_4_^+^ from wastewater through physical and biological processes. Similarly, He, Cao^39^ investigated the impact of different particle sizes on NH_4_^+^ removal efficiency, highlighting the importance of surface area and porosity in facilitating adsorption and microbial activity. However, this study observation extends beyond these static conditions to examine NH_4_^+^ behavior in response to dynamic wet/dry cycles. This approach is motivated by the recognition that SAT systems in real-world settings are subject to temporal variations in hydraulic loading and environmental and operational conditions due to seasonal variations in climate and hydrology. Incorporating wet and dry phases into the experimental design captures the transient responses of NH_4_^+^ removal mechanisms to changing moisture levels and oxygen availability. Furthermore, while previous studies have primarily focused on individual factors influencing NH_4_^+^ removal, such as pH, DO, and ORP, this analysis emphasizes the collective impact of these factors under dynamic conditions. Regression modeling and factorial interaction analysis elucidate the multifaceted relationships between NH_4_^+^ concentration and wet/dry parameters, providing a holistic understanding of the NH_4_^+^ dynamic’s fate and behavior in SAT systems. Moreover, the observations align with established theories and models of NH_4_^+^ behavior in wastewater treatment systems, particularly regarding the role of nitrification processes in reducing NH_4_^+^ concentration under aerobic conditions. The observed decrease in NH_4_^+^ concentration during periods of higher DO, pH, and ORP is consistent with the requirements for nitrification, wherein NH_4_^+^ is oxidized to NO_2_^−^ and then to NO_3_^−^ by nitrifying bacteria.

Additionally, the breakthrough curve for NO_3_^−^ through the sand column during the wet and dry periods over a 4 days column experiment with an initial concentration of 0 mg/L is also shown in Fig. 1—NO_3_. The observed fluctuations in NO_3_^−^ concentration within the sand column of the SAT system can be attributed to a complex interplay of environmental and operational factors influencing nitrogen transformation processes. Firstly, the initial NO_3_^−^ concentration increase during the first day, irrespective of wet or dry conditions, suggests active nitrification driven by favorable aerobic conditions. The subsequent decrease in NO_3_^−^ concentration on the second day, particularly during the wet period, may be linked to transient inhibitory factors impacting microbial activity. Potential factors contributing to this decline include nutrient depletion, variations in moisture content, oxygen availability, and unbalanced conditions, all of which can influence microbial metabolic rates and subsequently affect nitrification kinetics. Meanwhile, during the dry period, the continued increase in NO_3_^−^ concentration suggests sustained nitrification activity supported by favorable environmental conditions.

The sudden jump in NO_3_^−^ concentration at the beginning of the third day, following the dry period of the second day during the wet time, followed by a rapid drop during the same wet period, suggests a complex response of nitrogen transformation processes to changing environmental conditions within the system. One possible explanation for this phenomenon is the occurrence of rapid changes in microbial activity and nitrogen transformation rates in response to fluctuating DO and ORP conditions. The dry period preceding the second day may have resulted in conditions conducive to accumulating NH_4_^+^ within the sand column, potentially due to reduced microbial activity and nitrification rates under dry conditions. Upon reintroduction of moisture during the wet period on the third day, microbial activity could have been rapidly stimulated, leading to a surge in nitrification rates and subsequent conversion of accumulated NH_4_^+^ to NO_3_^−^, thus explaining the observed jump in NO_3_^−^ concentration. However, the sudden drop in NO_3_^−^ concentration during the same wet period may indicate a subsequent shift in environmental conditions that inhibit nitrification or promote denitrification. Factors such as fluctuations in oxygen availability and redox conditions can influence microbial community dynamics and nitrogen transformation processes where, in this period, the DO level is closer to zero mg/L, which becomes anaerobic conditions, and the ORP become in a reduction phase which highly promote the denitrifying bacteria to convert NO_3_^−^ to N_2_.

Furthermore, transient changes in microbial community composition, triggered by the transition from the dry to wet period, may have also contributed to the observed fluctuations in NO_3_^−^ concentration. Variations in moisture content can affect the accessibility of nutrients to microbial populations and alter the redox conditions within the system, influencing the balance between nitrification and denitrification processes. Subsequently, the near stabilization of NO_3_^−^ concentration throughout the dry period in the third and the fourth days suggests a dynamic equilibrium balance between nitrification and denitrification processes. This equilibrium state reflects the intricate balance between oxygen availability, nutrient supply, redox condition and microbial activity within the system, highlighting the importance of maintaining optimal conditions for nitrogen removal efficiency. Generally, the fluctuation in NO_3_^−^ concentration during the wet and dry periods likely reflects rapid responses of nitrogen transformation processes to changing environmental and operational conditions, including shifts in moisture content, microbial activity, oxygen availability, and redox conditions within the SAT system.

Moreover, the breakthrough curve for PO_4_^3−^ through the sand column during the wet and dry periods over a 4 day column experiment with an initial concentration of 20 mg/L is also shown in Fig. 1-PO_4_. The breakthrough curve (BTC) for PO_4_^3−^ demonstrates a consistently high removal capacity of PO_4_^3−^ from the synthetic wastewater in the sand column. Throughout the 4 days of the experiment, the sand column effectively reduced PO_4_^3−^ concentrations. On the first day, the concentration gradually increased during the wet period, remaining stable in the dry period, showcasing the sand's strong adsorption capacity for PO_4_^3−^. On the second day, a temporary concentration spike during the wet time, followed by a decrease to 0 mg/L in the dry period, suggested efficient removal. On the third day, PO_4_^3−^ concentrations stayed at 0 mg/L during wet and dry periods, indicating continuous effective removal. Day four saw a slight increase to 1 mg/L during the wet time, which was maintained in the dry period, likely due to some limitations in removal capacity at higher concentrations. Overall, these results confirm the sand column's exceptional ability to remove PO_4_^3−^ under various wet and dry conditions, relying on adsorption and other removal mechanisms to achieve low or negligible PO_4_^3−^ concentrations in the outflow.

The wet and dry cycles in soil aquifer treatment systems significantly impact synthetic wastewater pollutants’ removal and trendy behavior. We can better understand the mechanisms driving pollutant removal during wet and dry cycles by statistically correlating and examining the role relationships between wetting and drying factors (pH, DO, and ORP) and the dynamic changing in contaminants concentrations. The logistic regression model was constructed to assess the sufficiency of physiochemical parameters, namely pH, ORP, and DO, in describing the wet and dry conditions and their impact on pollutant removal capacity. The coefficients for the model are as follows: pH with p-value = 0.0134, ORP with p-value = 0.0499, and DO with p-value = 0.0388, which indicate statistically significant effects on the wet and dry conditions and their influence on pollutant removal capacity. pH, DO, and ORP are substantial predictors significantly impacting the condition represented by wet/dry periods.

**Figure 2:**
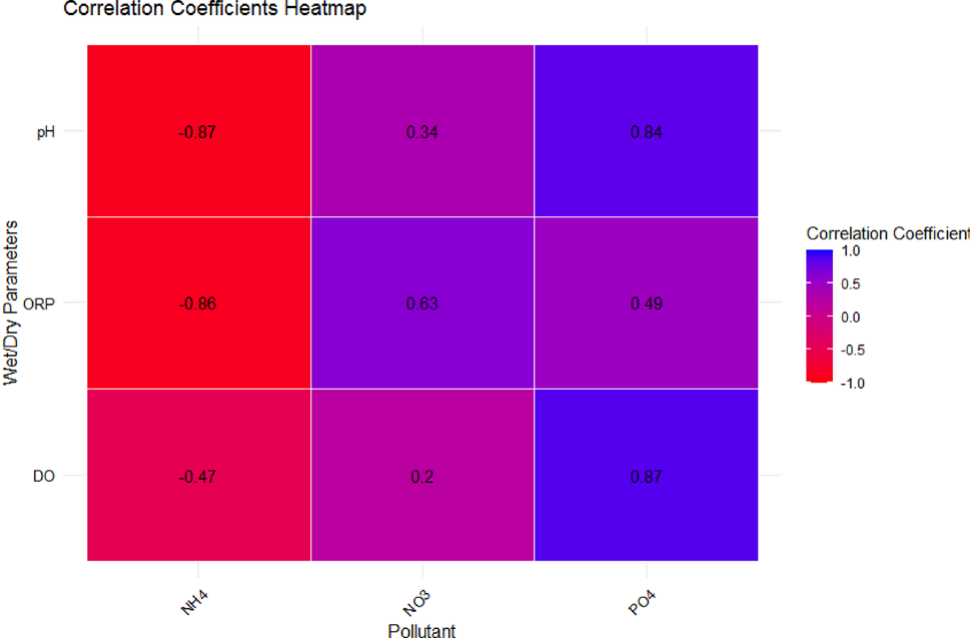
Correlation heatmap between NH_4_^+^, NO_3_^−^, PO_4_^3−^ and wet/dry parameters.

The relationship control roles between the nutrient concentration of NH_4_^+^, NO_3_^−^, PO_4_^3−^ and wet/dry conditions were explored by identifying the correlations, significant trends, and factorial interactions between the wet/dry factors from pH, DO, ORP and the NH_4_^+^, NO_3_^−^, PO_4_^3−^ concentration. The correlation analysis between NH_4_^+^, NO_3_^−^, and PO_4_^3−^ concentrations and wet/dry parameters was performed using a correlation heatmap, as shown in Fig. 2.

To analyze the interaction between NH_4_^+^ concentration and the wetting and drying conditions, we examined the relationship, correlations, and factorial interactions between NH_4_^+^ concentration and wet/dry key factors pH, DO, and ORP during wet and dry times. The regression model revealed significant relationships between NH_4_^+^ concentration and these factors, with p-values of 0.000053 for DO, 0.000031 for ORP, 0.01014 for pH, and 0.0575 for the wet/dry status. Additionally, the overall model was highly significant with a p-value of 1.64e−11, suggesting that the combined influence of these factors on NH_4_^+^ concentration is substantial. This analysis provides valuable insights into the complex relationships between NH_4_^+^ and the wet/dry variables during wet and dry periods, indicating their collective impact on NH_4_^+^ dynamics. The correlation analysis revealed strong negative relationships between NH_4_^+^ concentration and key wet/dry factors: − 0.87 with DO, − 0.47 with ORP, and − 0.86 with pH. These findings signify that as DO, ORP and pH values fluctuate during wet and dry conditions, NH_4_^+^ concentration experiences corresponding changes in the opposite direction. The high negative correlations emphasize the significant influence of these factors on NH_4_^+^ dynamics, underscoring their pivotal roles in shaping NH_4_^+^ behavior within the system and demonstrating the intricate interplay between environmental variables and pollutant concentration.

The negative observation correlations between NH_4_^+^ concentration and physiochemical factors reflect the complex interplay between environmental conditions, microbial activity, and NH_4_^+^ removal mechanisms. Through promoting aerobic conditions, pH optimization, and redox control, designers can effectively manipulate these factors to enhance NH_4_^+^ removal efficiency in SAT systems. The negative correlation between NH_4_^+^ concentration and DO levels can be attributed to the role of aerobic conditions in promoting NH_4_^+^ oxidation through nitrification. Nitrifying bacteria utilize oxygen to convert NH_4_^+^ into NO_2_^−^, followed by NO_3_^−^. As DO levels increase, the activity of these nitrifying bacteria is enhanced, leading to greater NH_4_^+^ removal efficiency.

Conversely, under anaerobic conditions with low DO levels, NH_4_^+^ may undergo alternative pathways, such as denitrification, reducing NH_4_^+^ removal rates. Also, the negative correlation between NH_4_^+^ concentration and ORP reflects the role of redox conditions in influencing NH_4_^+^ transformation pathways. Higher ORP values indicate more oxidizing conditions, which favor NH_4_^+^ oxidation through aerobic pathways. Conversely, lower ORP values indicative of reducing conditions may promote alternative NH_4_^+^ transformation processes, such as denitrification, leading to NH_4_^+^ accumulation in the system. Similarly, the negative correlation between NH_4_^+^ concentration and pH levels can be explained by the influence of pH on the equilibrium between NH_4_^+^ and its ionized form. In addition, pH could involve the activity of microbial communities within the system. Microbial processes such as nitrification are sensitive to pH levels. Nitrification is typically more efficient under neutral level conditions, while acidic conditions can inhibit nitrifying bacteria’s activity, leading to lower NH_4_^+^ oxidation rates and potentially higher NH_4_^+^ concentrations.

Also, the regression analysis examines the relationship between NO_3_^−^ concentration, wetting and drying conditions, and associated factors (pH, DO, and ORP). The p-values for the relationships between NO_3_^−^ concentration and the tested variables, along with the correlation coefficients, indicate these associations' statistical significance and strength. The relationship between NO_3_^−^ concentration and DO show a p-value of 0.1231, signifying no significant connection, with a weak positive correlation (r = 0.20). The relationship with ORP yielded a p-value of 0.0786, indicating no significant correlation, with a moderate positive correlation (r = 0.63). The relationship with pH had a p-value of 0.2847, implying no considerable association and a weak positive correlation (r = 0.34). Lastly, the relationship between NO_3_^−^ concentration and Wet/dry status had a p-value of 0.4872. The overall model's p-value was 0.373, revealing that the variations in NO_3_^−^ concentration during wet and dry periods were not strongly influenced by these factors, although some weak to moderate positive correlations were observed.

The lack of the statistical significance in these relationships suggests that other unmeasured factors might be affect at the system, influencing NO_3_^−^ dynamics in ways not captured by our experimental design. Sand adsorption, facilitated by the porous nature of the medium, has been documented as a mechanism for removing NO_3_^−^ from aqueous solutions^40^. Additionally, geochemical reactions involving minerals in the sand column may contribute to NO_3_^−^ attenuation through precipitation or mineral sorption^41^. Future research endeavors should consider investigating the role of sand adsorption and geochemical reactions in shaping NO_3_^−^ behavior and fate within similar systems.

In addition, the analysis of the interaction between PO_4_^3−^ concentration and wetting and drying conditions, including pH DO and ORP, the regression model revealed the relationships the analysis of the interaction between PO_4_^3−^ concentration and wetting and drying conditions, including pH, DO, and ORP, revealed significant relationships. The relationship between PO_4_^3−^ concentration and DO had a p-value of 0.00253, indicating a statistically significant relationship. The relationship between PO_4_^3−^ concentration and ORP also showed significance, with a p-value of 0.00208. The connection between PO_4_^3−^ concentration and pH had a p-value of 0.03276, suggesting a significant relationship but with a slightly higher p-value. The relationship between PO_4_^3−^ concentration and Wet/dry status was also substantial, with a p-value of 0.02919. The overall model had a very low p-value of 1.796e−08, confirming that the interactions between PO_4_^3−^ concentration and these factors significantly influenced the results, reflecting their combined impact on the removal of PO_4_^3−^. In the correlation analysis between PO_4_^3−^ concentration and wet/dry factors, the correlations were strong, with a correlation coefficient (r) of 0.871 between PO_4_^3−^ concentration and DO, 0.489 between PO_4_^3−^ concentration and ORP, and 0.840 between PO_4_^3−^ concentration and pH, emphasizing their substantial influence on PO_4_^3−^ removal from the system.

The principal component analysis was applied to understand the influence of dry and wet conditions on the concentrations of NH_4_^+^, NO_3_^−^, and PO_4_^3−^. PCA provides statistical descriptions, like eigenvalues and variables—the PCA eigenvalues, as shown in Table 3, represent the explained variance by each principal component.

**Table 3:** PCA statistical eigenvalues.

	Dim.1	Dim.2	Dim.3	Dim.4	Dim.5	Dim.6	Dim.7	Dim.8
Variance	4.097	2.446	1.125	0.607	0.322	0.249	0.107	0.047
% of variance	45.517	27.178	12.502	6.749	3.579	2.769	1.188	0.517
Cumulative %	45.517	72.695	85.197	91.947	95.526	98.295	99.483	100.00

In the eigenvalues analysis, the first principal component (Dim.1) explains the most significant variance, followed by Dim.2 and Dim.3. Cumulatively, the first three principal components explain a substantial portion (85.197%) of the total variance in the data. This indicates that these principal components effectively capture the most critical controlled variability in the system parameters.

While the PCA variables reveal the correlations of the WC, pH, DO, ORP, NH_4_^+^, NO_3_^−^, PO_4_^3−^ variables and dry/wet status with each principal component. Table 4 presents the variables correlation analysis for the first 3 principal components (Dim.1, Dim. 2, and Dim. 3). These principal components explain a high percentage of data, 85.197%, so they can effectively describe the whole system.

**Table 4:** PCA variables characteristics to the first third principal components.

Variable	Dim.1	Ctr	Dim.2	Ctr	Dim.3	Ctr
Status—Dry	0.460	5.162	− 0.860	30.263	0.012	0.013
Status—Wet	− 0.460	5.162	0.860	30.263	− 0.012	0.013
WC	− 0.542	7.168	0.678	18.816	0.032	0.091
pH	0.950	22.017	0.089	0.323	0.044	0.170
DO	0.910	20.235	0.320	4.195	0.041	0.148
ORP	0.721	12.700	0.248	2.507	0.361	11.569
NH_4_^+^	− 0.777	14.731	− 0.370	5.602	0.233	4.828
NO_3_^-^	0.053	0.069	0.107	0.465	0.926	76.288
PO_4_^3−^	0.723	12.755	0.430	7.566	− 0.278	6.878

The variable table presents the degree of correlations between the variable and the principal component and the contribution of each variable to the principal component. The (ctr) values represent the contributions of each variable to the principal components. The first principal component (Dim.1) shows a strong positive correlation with pH, DO, and ORP, while NH_4_^+^ has a strong negative correlation. This suggests that Dim.1 captures variability related to parameters associated with pollution levels in the system. The second principal component (Dim.2) is positively correlated with NH_4_^+^ and PO_4_^3−^ and negatively correlated with pH, DO, and ORP. This component may represent variations related to nutrient concentrations and water chemistry.

The variables Status—Dry and Status—Wet exhibit opposite correlations along Dim.1, indicating that they are associated with different patterns of concentration parameters. This suggests that dry and wet conditions influence the overall composition and concentrations of pollutants in the SAT system. Moreover, the strong correlation between Status—Dry and Status—Wet with the first principal component suggests that these conditions significantly affect the concentration parameter variability. Also, the opposite correlations of Status—Dry and Status—Wet along Dim.1 imply that dry and wet conditions may have contrasting effects on the concentrations of pollutants.

Figure 2 represents the PCA contributions of variables to the first two principal components (Dim.1 and Dim.2), providing insights into their relative importance in explaining the variability in the concentration trends. As shown in Fig. 2, the variable of WC, pH, DO, ORP, NH_4_^+^, NO_3_^−^, and PO_4_^3−^ and the status wet/dry indicator, positioned based on its contribution to the variability explained by Dim.1 and Dim.2 principal components. Variables with more immense contributions have more significant coordinates along Dim.1 and Dim.2, suggesting their more substantial influence on the overall variability observed in the system.

**Figure 3:**
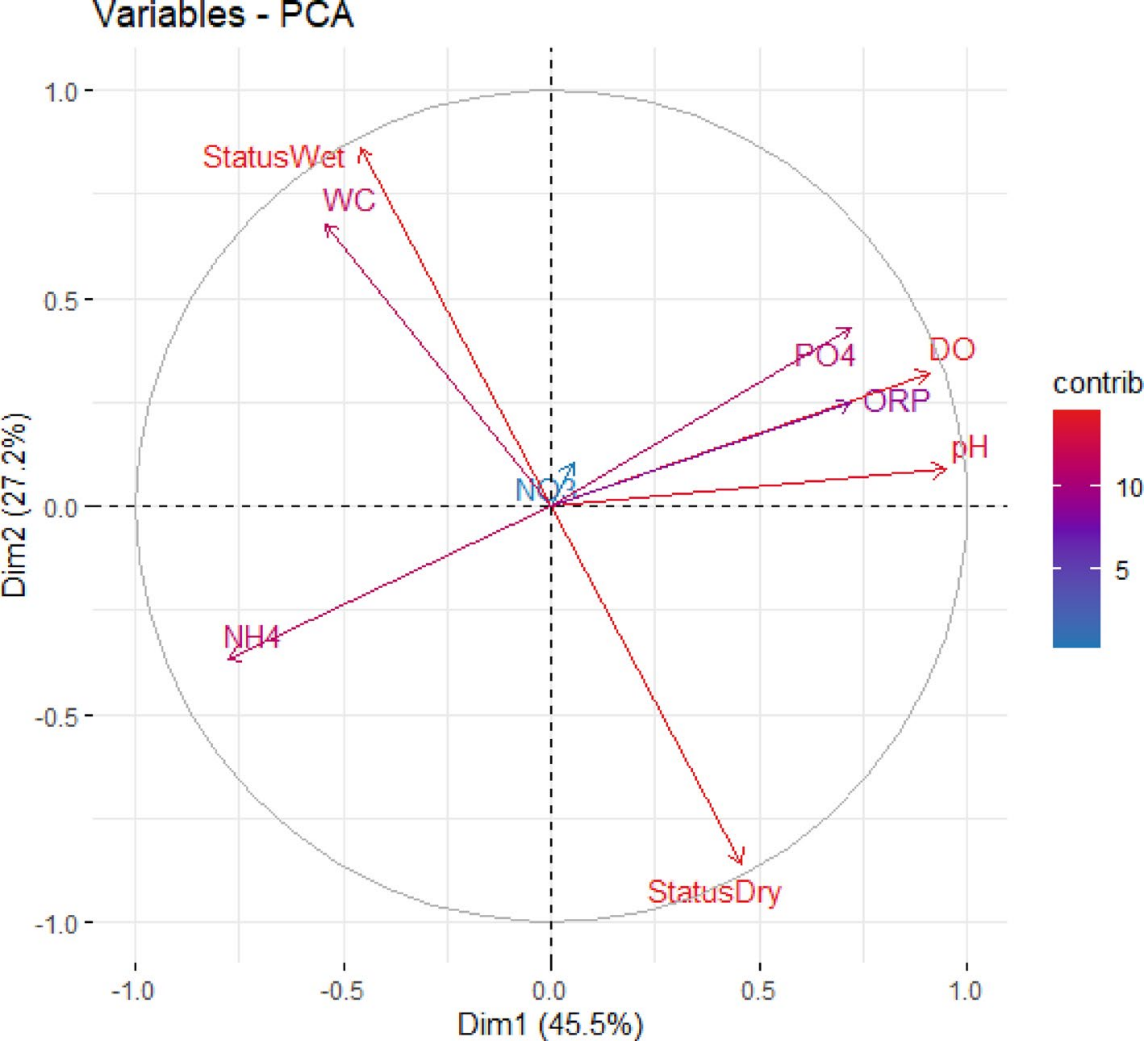
PCA contributions interaction of variables to the first two principal components.

Both Status-Dry and Status-Wet have notable contributions, suggesting their significance in defining the primary source of variability in the system. Their opposite correlations indicate their contrasting effects on concentration parameters. pH, DO, and ORP demonstrate substantial contributions to Dim.1, indicating their importance in shaping the whole system variations. The PCA show that these parameters have high loadings on Dim.1, reflecting their strong correlations with this principal component. NH_4_^+^, NO_3_^−^, and PO_4_^3−^ contribute significantly to the overall variability, as suggested by their loadings on Dim.1 and Dim.2 in the PCA Fig. 3.

Overall, the variables with higher contributions, such as status dry, status wet, pH, DO, and ORP, play crucial roles in shaping concentration variability, as evidenced by their significant loadings on the principal components. The contrasting effects of dry and wet conditions, as indicated by Status-Dry and Status-Wet, highlight the importance of wet and dry conditions and factors in influencing NH_4_^+^, NO_3_^−^, and PO_4_^3−^ concentrations dynamics.

Dry conditions lead to decreased NH_4_^+^ concentration but may not necessarily affect NO_3_^−^ or PO_4_^−^ significantly. While Dim.1 is negatively correlated with NH_4_^+^, the interpretation for Dim.2 positively correlates with NH_4_^+^ and PO_4_^−^. Dry conditions may have a more significant impact on NH_4_^+^ than NO_3_^−^ or PO_4_^−^. Wet conditions could potentially influence both NH_4_^+^ and NO_3_^−^. Dim 2 shows a positive correlation with NH_4_^+^ and NO_3_^−^. This analysis suggests that dry and wet conditions have contrasting effects on pollutant concentrations in the SAT system. Dryness seems linked to higher potential pollution levels, while wetness might favor lower pollution. The impact of dry and wet conditions on NH_4_^+^ is more evident, with dry conditions potentially leading to lower NH_4_^+^ concentration. while wet conditions effects on NO_3_^−^ and PO_4_^−^.

**Figure 4:**
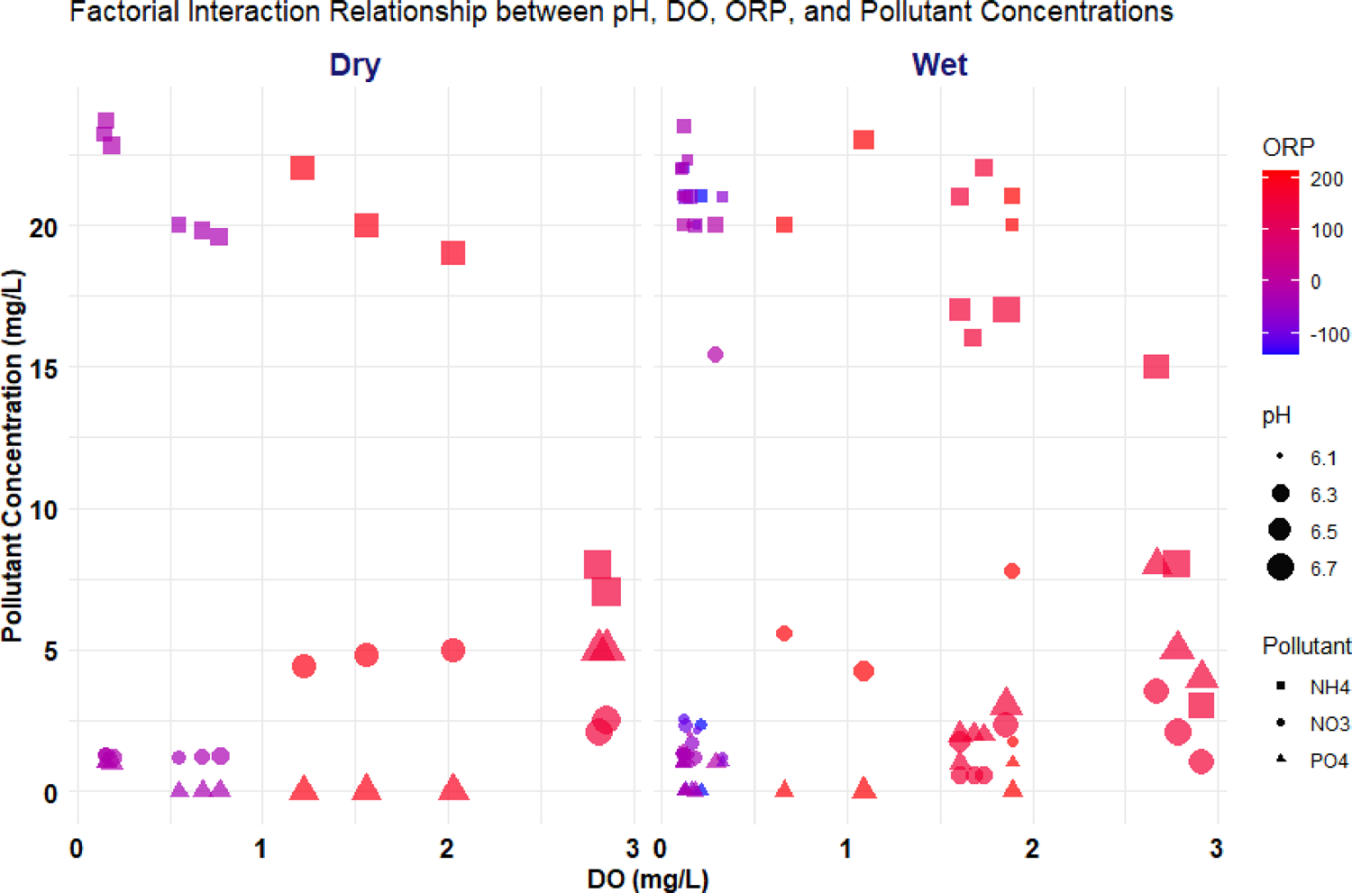
Factorial interaction relationship between nutrients concentration (NH_4_^+^, NO_3_^−^, PO_4_^3−^) with wet and dry parameters.

The relationships investigation used the factorial interaction analysis to explore the dynamics between nutrient pollutants (NH_4_^+^, NO_3_^−^, PO_4_^3−^) and environmental, operational key factors, specifically the wet and dry conditions of pH, DO, and ORP, and their collective impact on pollutant concentrations. Figure 4 displays the significant factorial interactions between the concentrations of NH_4_^+^, NO_3_^−^, PO_4_^3−^ and the wetting and drying conditions.

To analyze the factorial interaction between NH_4_^+^ concentration and the wetting and drying conditions, the interaction relationships trend examined between NH_4_^+^ concentration and critical parameters such as pH, dissolved oxygen (DO), and oxidation–reduction potential (ORP) during both wet and dry times as shown in Fig. 4. The factorial interaction analysis based on the regression model highlights the intricate relationships between NH_4_^+^ concentration and wet/dry parameters, specifically DO, pH, and ORP, during both wet and dry periods. The results indicate that as DO, pH, and ORP increase during wet and dry times, there is a corresponding decrease in NH_4_^+^ concentration. This finding aligns with the correlation analysis, which demonstrated strong negative correlations between NH_4_^+^ and these parameters. The alignment between the observed reduction in NH_4_^+^ concentration with increasing DO, pH, and ORP also corresponds to the requirements for nitrification degradation in which NH_4_^+^ is converted into nitrite (NO_2_^−^) and then nitrate (NO_3_^−^), which ultimately results in the removal of ammonium from the system^42^. This process highly depends on the availability of dissolved oxygen (DO), pH, and oxidation–reduction potential (ORP). Adequate DO levels support the activity of nitrifying bacteria, while specific pH conditions and ORP values are conducive to their growth and nitrification activity^43^. Together, these results confirm that the increase in DO, pH, and ORP exerts a controlling effect on reducing NH_4_^+^ concentration, emphasizing the significant influence of these environmental factors on NH_4_^+^ dynamics within the system.

In addition, the correlation between increasing DO, pH, and ORP and decreasing NH_4_^+^ concentration suggests a strong relationship between environmental conditions such as the role of nitrification processes and NH_4_^+^ removal kinetics, which is fundamental to existing theories and models of NH_4_^+^ behavior in SAT systems. Our findings validate these models empirically, demonstrating their relevance in predicting NH_4_^+^ dynamics under dynamic wet/dry conditions. Furthermore, these observations highlight the importance of considering the temporal variability of environmental factors in SAT system operation and optimization. While existing theories often focus on steady-state conditions, our results emphasize the dynamic nature of NH_4_^+^ removal processes, which are influenced by fluctuations in moisture levels, oxygen availability, and microbial activity. By integrating our empirical findings with existing theoretical frameworks, we contribute to refining and validating models describing NH_4_^+^ behavior in SAT systems. This investigation underscores the need for dynamic modeling approaches that account for temporal variations in environmental parameters, providing more accurate predictions of NH_4_^+^ removal efficiency and system performance.

As determined by the regression model, the factorial interaction relationships between NO_3_^−^ concentration and wet/dry parameters highlight these factors’ roles during wet and dry periods. There's a general trend where increasing DO, pH, and ORP during wet and dry times correspond to an increase in NO_3_^−^ concentration. This observation aligns with the positive correlation found in the correlation analysis, indicating that these factors positively influence NO_3_^−^ concentration. This behavior can be attributed to nitrification processes converting NH_4_^+^ to NO_3_^−^, where NO_3_^−^ represents the final oxidization product. Conversely, the decrease in NO_3_^−^ concentration is associated with denitrification, which is influenced by decreasing DO and ORP levels, as both indicate reduced oxygen availability in the system^43^. Denitrification reduces NO_3_^−^ to N_2_ gas, contributing to a decrease in NO_3_^−^ concentration.

The factorial interaction relationships based on the regression model highlighted the roles of PO_4_^3−^ concerning DO, pH, and ORP during wet and dry times. It was observed that an increase in DO, pH, and ORP during both wet and dry conditions led to a rise in PO_4_^3−^ concentration. This trend aligned with the positive correlations revealed in the correlation analysis, further emphasizing the influential roles of these factors in controlling PO_4_^3−^ concentrations. Additionally, the likelihood of PO_4_^3−^ precipitating with cations such as Fe^2+^ or Ca^2+^ contributed to the increased removal capacity for PO_4_^3,44^. This interaction and the potential precipitation processes played a significant role in removing PO_4_^3−^ from the system, further corroborating the positive influence of these factors on PO_4_^3−^ removal.

Phosphate precipitation involves the formation of insoluble phosphate compounds through reactions with divalent cations in the water matrix^45^. Within SAT systems, this process occurs as treated wastewater infiltrates through the soil matrix, interacting with naturally occurring cations. Under favorable conditions, phosphate ions react with cations such as Fe^2+^, Al^3+^, Mg^2+^ or Ca^2+^ to form precipitates, thereby reducing phosphate concentrations in the treated effluent^46^. The precipitation process is influenced by several factors, including pH, temperature, and the concentrations of phosphate and cations. In SAT systems, soil pH plays a critical role in governing phosphate speciation and solubility, thereby affecting the potential for precipitation reactions^47^. Additionally, variations in redox potential (ORP) within the soil matrix and the presence of other ions can also impact the kinetics and extent of phosphate precipitation. By manipulating parameters such as soil pH, ORP, or cation concentrations, it becomes possible to promote the formation of insoluble phosphate precipitates, thereby improving overall treatment performance.

**Figure 5:**
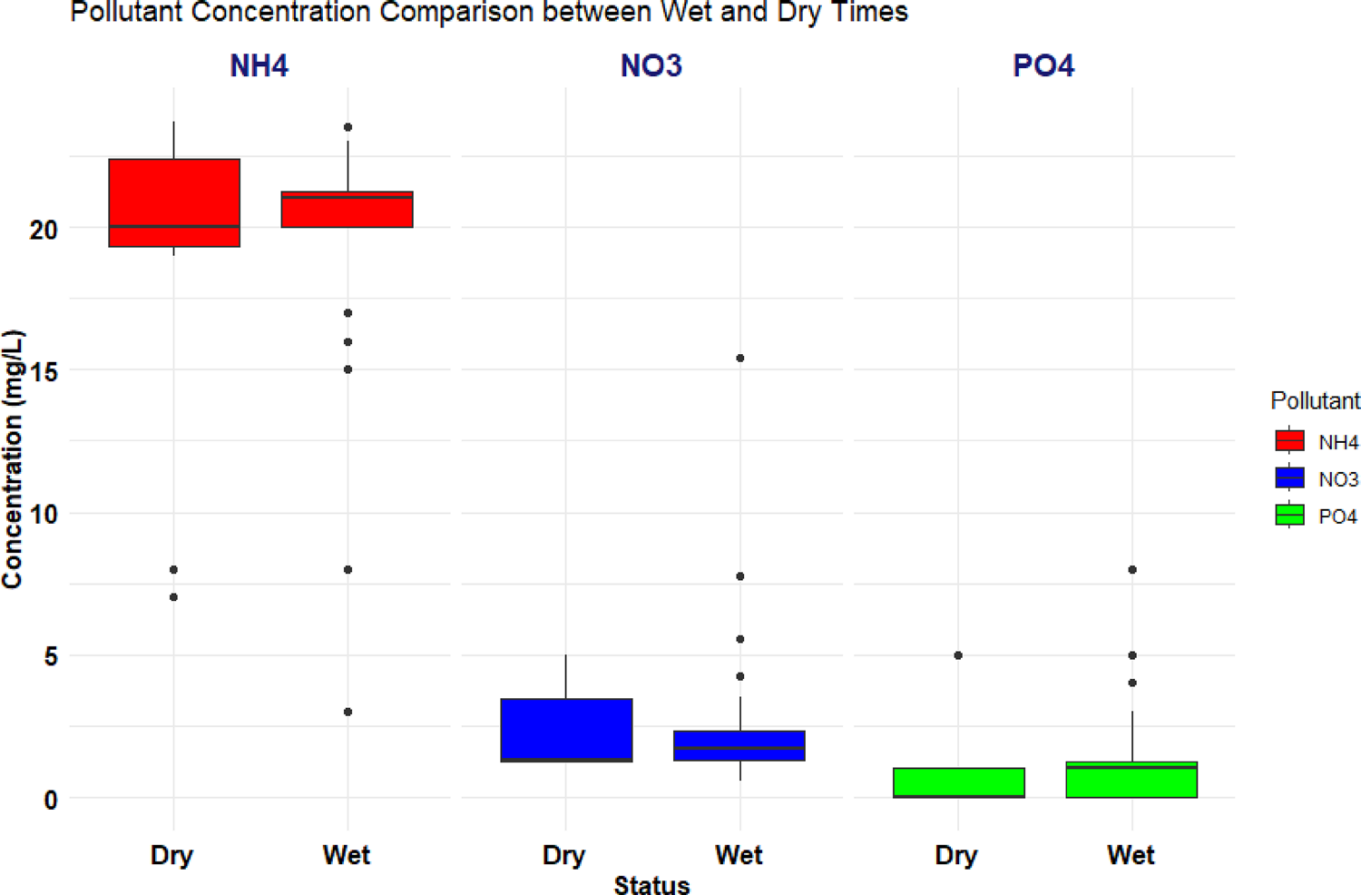
Box plot comparison between nutrients (NH_4_^+^, NO_3_^−^, PO_4_^3−^) concentrations under wet and dry times.

To summarize the effect of wet and dry periods on the NH_4_^+^, NO_3_^−^, and PO_4_^3−^ concentrations, the comparison box plots were used to visualize the favorable period for removal conditions, as shown in Fig. 5.

The box plot was used to visualize the favorable period for NH_4_^+^ removal conditions. As shown in Fig. 5, the box plot analysis aimed to understand the impact of wet and dry periods on NH_4_^+^ concentration by visualization comparisons. The results indicate a slight difference in mean NH_4_^+^ concentration between wet and dry times, with the dry period exhibiting slightly lower values. This suggests that NH_4_^+^ removal conditions are favorable during the dry period, aligning with Abel^48^ and Ma, Yang^49^. In summary, the dry period is associated with a slightly lower NH_4_^+^ concentration, implying better NH_4_^+^ removal during this time compared to the wet period. Additionally, the box plot analysis used to assess the impact of wet and dry periods on NO_3_^−^ concentration revealed that the mean NO_3_^−^ concentration during dry times was generally lower than that during wet times, which aligns with Ma, Yang^49^, and Sallwey, Jurado^50^. This suggests that dry periods are more favorable for NO_3_^−^ removal conditions. In addition, the analysis of wet and dry periods’ effects on PO_4_^3−^ concentrations, as visualized by box plots, revealed that, in general, the mean PO_4_^3−^ concentration during dry times was lower than during wet times, which is aligned with Wang, Lin^51^. This indicates that dry periods were more favorable for removing PO_4_^3−^ from the system. The observations align with the high removal capacity for PO_4_^3−^ and the influence of factors such as DO, pH, ORP, and potential precipitation processes, emphasizing the effectiveness of the sand column in reducing PO_4_^3−^ concentrations, particularly during dry conditions.

Table 5 compares the removal performance rate for Total N_2_ as (NH_4_^+^ and NO_3_^−^) and PO_4_^3−^ under wet and dry conditions in the SAT system.

**Table 5:** Synthetic wastewater pollutants removal amount and capacities.

	Wet q_total_ (mg)	Dry q_total_ (mg)	Total q_total_ (mg)	Total infiltrated mass (mg)	Wet-R%	Dry-R%	Total R%
Total N_2_ (NH_4_^+^ + NO_3_^−^)	18.33	21.56	39.89	70 as NH_4_^+^	73%	69%	43%
PO_4_^3−^	1.53	1.34	2.87	40	96%	97%	93%

The findings from comparing the removal capacity rate of Total N_2_ (NH_4_^+^ + NO_3_^−^) and PO_4_^3−^ under wet and dry conditions reveal a notable overall elimination performance for both pollutants, indicating the effectiveness of the SAT system in removing contaminants from infiltrated synthetic wastewater. The high removal rates of 93% for PO_4_^3−^ and 43% for Total N_2_ demonstrate the system's capability to reduce concentrations significantly. Moreover, while there is a slight difference in Total N_2_ removal efficiencies between wet (73%) and dry (69%) conditions, indicating a slightly higher efficiency during wet conditions, the overall performance remains effective across both wet and dry conditions.

This study bridges the gap between experimental observations and theoretical frameworks, shedding light on the complex dynamics of SAT systems. By integrating insights from the literature, it can offer new perspectives on pollutant removal mechanisms and system optimization strategies. Furthermore, these findings underscore the importance of considering both hydraulic and biological processes in effectively designing and operating SAT systems. These experimental results reveal fluctuations in pollutant concentrations during wet and dry cycles, highlighting the dynamic nature of pollutant removal in SAT systems. By comparing our observations with findings from the literature, particularly studies on bioaccumulation in intermittent sand filters (ISFs)^52^ and the impact of clogging on system performance^53^ and^54^, we can elucidate the underlying mechanisms driving these fluctuations. For instance, the observed decrease in pollutant concentrations during dry periods may be attributed to enhanced adsorption and microbial activity, consistent with predictions from theoretical models like the BIO_PORE model and the Cartridge Theory.

The observed nutrient transformations, mainly removing NH_4_^+^, NO_3_^−^, and PO_4_^3−^, during wet and dry cycles significantly affect ecosystem health. The efficient removal of these pollutants from synthetic wastewater within the SAT system highlights its potential to mitigate nutrient pollution in groundwater and surface water bodies. However, it is essential to consider the potential ecological impacts of nutrient transformations within the system. For instance, releasing NO_3_^−^ and PO_4_^3−^ into groundwater could lead to groundwater contamination and eutrophication of receiving water bodies, impacting aquatic ecosystems. Furthermore, the interactions between nutrients and soil microbial communities may influence nutrient cycling processes and soil fertility. Understanding these ecological dynamics is crucial for assessing SAT systems' long-term sustainability and compatibility with surrounding ecosystems.

While these laboratory-scale experiments provide valuable insights into nutrient dynamics within SAT systems, they are considered as the foundation for optimizing real-world applications. It's crucial to consider the scalability of our findings to larger, real-scale SAT systems. Variations in hydraulic loading rates, soil characteristics, and environmental conditions across SAT installations can influence nutrient transformation processes. Nevertheless, the fundamental mechanisms uncovered in this study, such as nutrient adsorption onto soil particles, microbial-mediated transformations, and the linking of the physiochemical parameters with the operational issues to explore the fate and behavior of nutrient pollutants, remain relevant to real-world SAT systems. Indeed, the scalability of findings from lab-scale studies to field applications is essential. It acknowledges the complexities and challenges of translating theoretical knowledge into practical solutions. By recognizing the significance of lab-scale experimentation, researchers can bridge the gap between theory and application, paving the way for the effective optimal design and implementation of SAT systems on a larger scale. Future research efforts should focus on validating our findings in field-scale settings and optimizing SAT system designs to maximize nutrient removal efficiency while minimizing ecological impacts.

## Conclusion

A comprehensive investigation of a simulated soil aquifer treatment (SAT) system in the laboratory-scale study has unveiled the intricate interplay between wet and dry cycles and physicochemical parameters, shedding light on their profound influence on pollutant removal dynamics. The observed fluctuations in soil water content, pH, dissolved oxygen, and oxidation–reduction potential during alternating wet and dry phases underscore their pivotal roles in shaping diffusion, adsorption, and microbial activity within the system. Noteworthy findings include the discernible impact of these wet and dry conditions on ammonium (NH_4_^+^), nitrate (NO_3_^−^), and phosphate (PO_4_^3−^) removal efficiencies. Specifically, dry periods consistently demonstrated higher removal rates for NH_4_^+^, NO_3_^−^, and PO_4_^3−^, signifying the importance of these conditions in optimizing pollutant removal within SAT systems. Additionally, the logistic regression model affirmed the statistical significance of pH, DO, and ORP in predicting wet/dry conditions, further emphasizing their indispensable roles. The study's outcomes contribute nuanced insights into the complexities of SAT systems, providing valuable data for system optimization and the development of sustainable wastewater treatment practices. These results enhance our understanding of pollutant dynamics and carry practical implications for designing and implementing efficient SAT systems in diverse environmental contexts.

## Data Availability

All improved and analyzed datasets over the current study are available under a reasonable request from the corresponding author.
